# Quality of Life in the First Year after Ischemic Stroke Treated with Acute Revascularization Therapy

**DOI:** 10.3390/jcm11113240

**Published:** 2022-06-06

**Authors:** Aboudou Matinou Do Rego, Gauthier Duloquin, Marie Sauvant, Simon Amaral, Quentin Thomas, Hervé Devilliers, Yannick Béjot

**Affiliations:** 1 INSERM CIC-1432 Clinical Investigation Center—Clinical Epidemiology, Dijon University Hospital, 21000 Dijon, France; mattydov@gmail.com (A.M.D.R.); herve.devilliers@chu-dijon.fr (H.D.); 2 Dijon Stroke Registry, EA7460, University of Burgundy, 21000 Dijon, France; gauthier.duloquin@chu-dijon.fr (G.D.); marie.sauvant@chu-dijon.fr (M.S.); simon.amaral@chu-dijon.fr (S.A.); quentin.thomas@chu-dijon.fr (Q.T.); 3 Neurology Department, Dijon University Hospital, 21000 Dijon, France; 4 Internal Medicine and Systemic Diseases Unit, Dijon University Hospital, 21000 Dijon, France

**Keywords:** acute ischemic stroke, patient reported outcomes, quality of life, revascularization therapy, thrombolysis, mechanical thrombectomy

## Abstract

(1) Background: we aimed to describe the disease-specific quality of life (QoL) of ischemic stroke patients treated with acute revascularization therapy, its evolution from 6 months to 12 months, and associated factors. (2) Methods: QoL was assessed with the SS-QoL in consecutive patients treated with either intravenous thrombolysis (IVT) and/or mechanical thrombectomy (MT). Variables associated with QoL scores and its evolution were studied using multivariate mixed models, and interaction with time. Analyses were performed in four domains of SS-QoL: self-care, mobility, mood, and social roles. (3) Results: Among the 501 included patients (mean (sd) age 68.9 (14.5), 49% women), lower post-stroke QoL was independently related to lower level of school education, prestroke mRS > 2, and 24 h NIHSS score > 4. Independent predictors of unfavorable evolution of QoL over time were age <75 years (Mobility *p* = 0.0194 and Mood *p* = 0.0015), NIHSS score ≤ 4, (Self-care *p* = 0.0053 and Mood *p* = 0.0048), and modified Rankin Scale score ≤ 2 (Social roles, *p* = 0.0006). Revascularization therapy had no significant effect on the QoL scores, but patients treated with MT (alone or as bridging therapy) had significantly greater improvement in mobility score between 6 and 12 months than patients treated with IVT alone (*p* = 0.0072). (4) Conclusion: QoL evolution over one year had only slight variation and was associated with the modalities of acute treatment, age, and stroke severity.

## 1. Introduction

More than 110,000 new cases of stroke are reported each year in France, and the absolute number is on the rise due to the aging population [1]. While prevention remains a major challenge, considerable progress was made in acute stroke treatment over the past years, namely revascularization therapy including either intravenous thrombolysis (IVT) and/or mechanical thrombectomy (MT). This contributed to a better post-stroke outcome of patients with a decrease in case-fatality and a higher number of stroke survivors, thus involving significant impact on healthcare system resources [2,3]. Despite these improvements, stroke remains a disabling disease. The quality of life (QoL) of patients in the post-acute phase of ischemic stroke is still very much affected, and its evolution over time has been shown to depend on many factors such as the area of the brain affected [4], the delay in treatment administration, and the functional outcome of patients [5,6]. Although the effectiveness of therapeutic strategies in acute ischemic stroke in reducing motor disability has been widely demonstrated by randomized clinical trials [7,8], their impact on patients’ QoL remains poorly evaluated. Few studies addressed this issue, most of them being limited to an evaluation of patients after a short period of three months following stroke [4,5,6,9], and there is very little data on QoL after six months among patients treated by IVT, MT or combined treatment [10,11,12]. The use of a disease-specific questionnaire may allow investigating specific concerns of stroke patients with a better sensitivity to change than generic questionnaires. The Stroke-Specific Quality-of-Life (SS-QoL) scale is a specific instrument that assesses the QoL of stroke patients. An adaptation and validation of the initial version was proposed by a study carried out by our team [13]. This instrument showed satisfactory psychometric properties in terms of validity, reliability, and responsiveness [11,13]. It was designed to be self-administered or even administered by telephone [14] with a good reliability except in aphasic patients. It differs from other instruments (e.g., Stroke Impact Scale [SIS]) by its shorter administration time [15] and after several evaluations is recognized as one of the most comprehensive measures of quality of life after stroke [11]. For patients and clinicians, knowledge of the determinants of the QoL in stroke survivors who received revascularization therapy would help to predict the evolution of the impact of the disease on patients’ lives.

The aim of our study was to describe variables associated with disease-specific SS-QoL scores and its evolution between 6 and 12 months in stroke patients treated with IVT and/or MT.

## 2. Materials and Methods

### 2.1. Patients and Design

The Prognosis After Revascularization therapy in the Dijon Ischemic Stroke Evaluation (PARADISE) Study was a single-center prospective observational cohort study conducted from January 2016 to June 2019 at Dijon University Hospital (NCT02856074). Consecutive patients aged 18 years or older were included if they had acute ischemic stroke treated with revascularization therapy (either IVT and/or MT). Patients received information about the study and gave their oral consent to participate according to the French legislation. The study was approved by a French ethic committee (CCP Est I, IRB number: 2015-A01664-45).

### 2.2. Data Collection and Patients’ Follow-Up

At inclusion, several data were collected including sociodemographic characteristics (sex, age, occupation, marital status), medical history including vascular risk factors, and prestroke treatments. Premorbid handicap was assessed using the modified Rankin scale (mRS) score. Stroke severity was quantified at admission and at 24 h by the National Institutes of Health Stroke Scale (NIHSS) score. Patients were followed by a phone contact at 3 months, a face-to-face visit with a stroke-trained neurologist at 6 months and a last phone contact at 12 months. During each visit the functional status of patients was assessed by the mRS score. Patients’ QoL after stroke was evaluated at 6 months and 12 months with the French version of the SS-QoL, a 49-item scale in 12 domains, namely Self-care, Fatigue, Mobility, Mood, Social Role, Family Role, Language, Character, Productivity/Work, Memory/Concentration, Vision, and Upper Extremity Function. This scale had been cross-culturally validated into the French language [13]. The score in each domain was transformed into a score from 0 (worst QoL) to 100 (best possible QoL). Patients with severe aphasia or dementia, who were not able to complete the questionnaire, were not assessed. For the present study, we analyzed data from subjects enrolled in the PARADISE study for whom the SS-QOL score was recorded at 6 months and/or 12 months.

### 2.3. Statistical Analyses

Categorical variables were described as frequencies and percentages, quantitative variables as means ± standard deviation. We compared the characteristics of patients with SS-QoL score data available at 6 or 12 months to those of patients who did not complete the questionnaire using Fisher exact test, Chi-2, or a Mann-Whitney test, when appropriate. The QoL scores were described as well as their respective evolution between 6 months and 12 months. The relationships between each independent variable and QoL scores were studied in a linear random-effects model (mixed model) that allowed repeated measurements to be taken into account, even in the case of missing data at either of the two measurement periods. QoL score in a given domain was modelled as the dependent variable. Thanks to this approach, we modelled the effect of each independent variable, as well as the effect of time (6 or 12 months), and of the time * variable interaction. Briefly, random-effects model allows one to take into account the correlation between 6- and 12-month measurements for a given patient. A significant time effect indicated a change in the quality-of-life score between 6 and 12 months, a significant variable effect indicated an overall difference in QoL between the two groups, and a significant time * variable interaction an increase (or a decrease) in the QoL difference between the two groups between 6 and 12 months. Independent variables with a significant effect on QoL in the previous analysis were proposed for entry into a multivariate mixed model with a manual backward selection procedure and a variable exit threshold set at 0.10. The final model fit was checked according to Akaike Information Criterion (AIC) and Bayesian Information Criterion (BIC). These analyses were performed separately for 4 domains of the SS-QoL: Self-care, Mobility, Mood, and Social Roles. For patients for whom QoL data were missing at only one of the two time points (6 or 12 months), the available data were included in the analysis. A sensitivity analysis was performed on the complete data. Statistics were performed using SAS^®^ 9.3 software (Cary, NC, USA).

## 3. Results

### 3.1. Patients’ Characteristics

**Figure 1:**
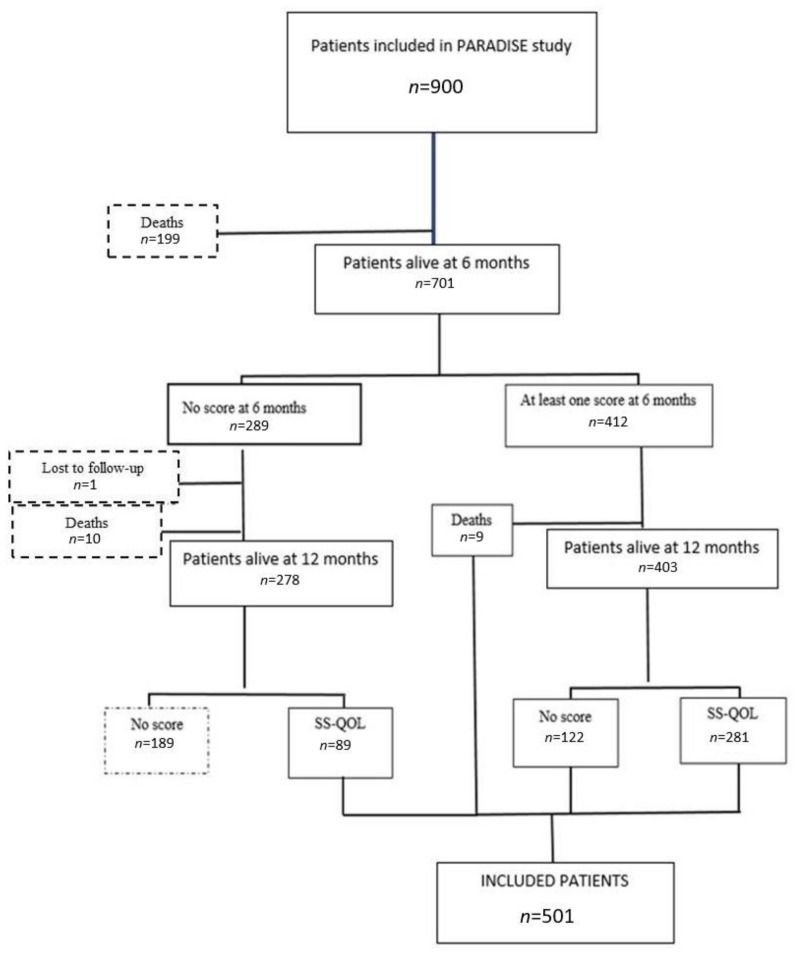
Participant selection flowchart.

Between January 2016 and June 2019, 900 patients were included in the PARADISE study. SS-QoL data were missing for 399 (44%) patients, 199 of whom died before 6 months. Among the 501 patients included in the present analysis, SS-QoL data were available at both time points (6 and 12 months) for 281 (56%) patients, only at 6 months for 131 patients (26%) patients, and solely at 12 months for 89 patients (18%) (Figure 1).

Patients who completed the SS-QoL questionnaire were significantly younger (mean age ± SD: 68.9 ± 14.5 vs. 76.7 ± 13.2 years, *p* < 0.001), less often institutionalized before stroke (5% vs. 11%, *p* < 0.001), and treated significantly more often with IVT alone (55% vs. 39%, *p* < 0.001) compared to patients who did not (Table 1). The included participants had a lower NIHSS score at 24 h (mean (SD): 5.5 ± 5.4 vs. 13 (8.5), *p* < 0.0001). Among the 501 analyzed patients, 247 (49%) were women, 270 (55%) received IVT only, 121 (24%) MT only, and 104 (21%) combined therapy. The median (IQR) age was 70 (60–80).

### 3.2. Description and Time Evolution of SS-QoL Scores

In the studied domains of QoL, on a scale ranging between 0 and 100 (100 being the best QoL), median scores ranged between 80 to 100 at 6 months and between 90 and 100 at 12 months (Table 2). The best scores were observed in the Self-care domain (median at 6 and 12 months: 100) and the worst in the social roles domain (median at 6 and 12 months at 80 and 90, respectively). There was a trend toward improvement in QoL scores between 6 and 12 months for the self-care, mobility, and social roles domains (mean (SD) improvement +1.8 (13.6), +1.9 (17.7), and +3.8 (28.7), respectively), and a small decrease in the mean score for the mood domain of −0.26 (21.0).

### 3.3. Time-Adjusted Bivariate Analysis

There was significant improvement in QoL scores from 6 to 12 months in all 4 domains studied in all models (Table 3). Female sex, age > 75 years, school level less than high school diploma, hypertension, renal failure, and pre-stroke mRS score > 2 were significantly associated with lower scores in all explored domains except mood (*p* < 0.05 in self-care, mobility, and social roles domains). Atrial fibrillation was associated with a lower QoL in the self-care (*p* = 0.0017) and mobility (*p* = 0.0024) domains. Underweight and obstructive sleep apnea (OSA) syndrome were associated with lower scores for self-care (*p* = 0.0247 and *p* = 0.0312, respectively). Patients who received combined treatment (IVT + MT), and those with a higher NIHSS score at 24 h (between 5 and 20) had a significantly lower QoL in the mood domain than those who received IVT alone, or those with a 24-h NIHSS score ≤ 4 (*p* = 0.001 and *p* < 0.0001, respectively).

**Figure 2:**
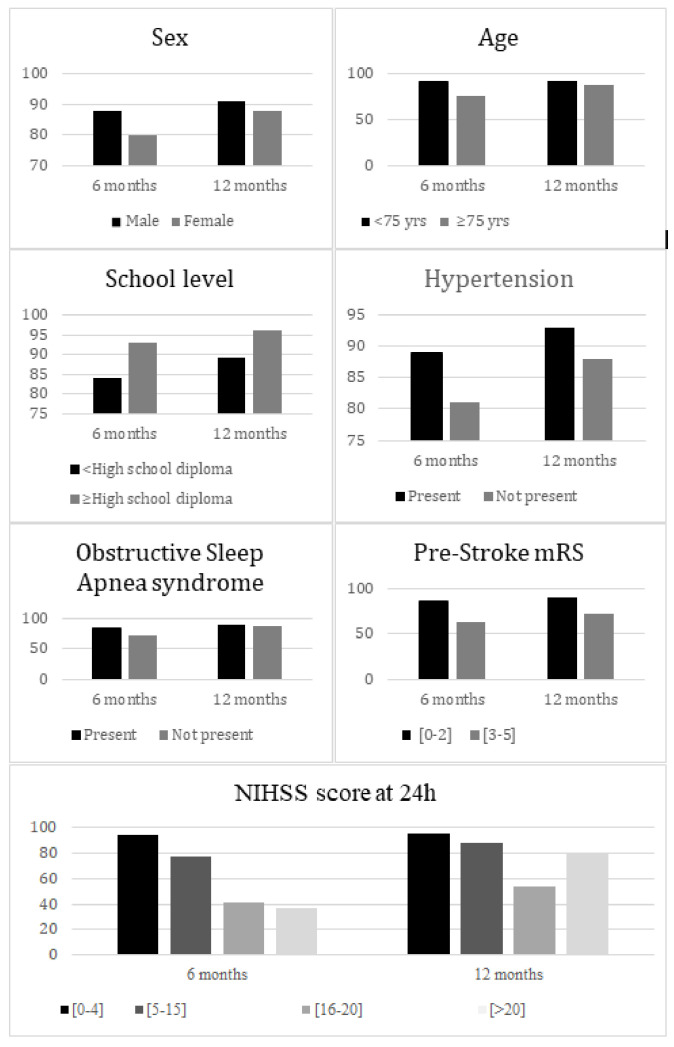
Mean self-care score at 6 months and 12 months according to patients’ characteristics.

The observed difference in self-care scores narrowed significantly over time between groups defined by a NIHSS score at 24 h ≤ 4 vs. 5–15, and age over versus below 75 years (interaction *p* < 0.0001 and 0.0189, respectively, Figure 2).

**Figure 3:**
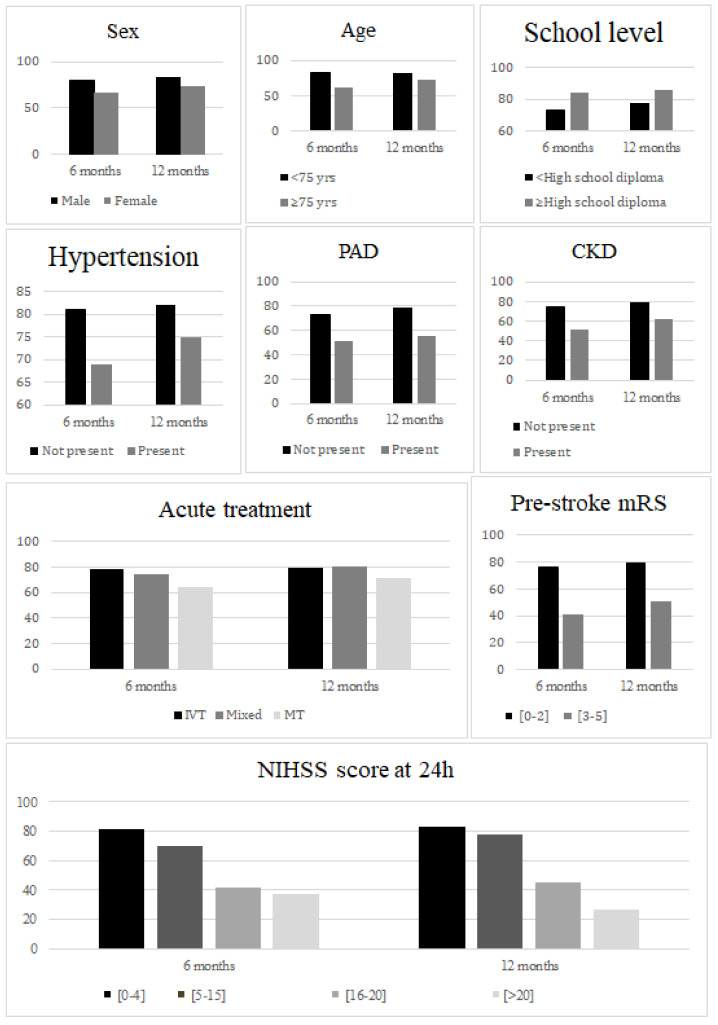
Mean mobility score at 6 months and 12 months according to patients’ characteristics. *PAD, peripheral artery disease; CKD, chronic kidney disease*.

For mobility scores, the observed difference was significantly reduced over time between groups defined by age ≥ 75 or <75 years, by treatment with IVT alone versus combined therapy (interaction *p* 0.0464 and 0.0484, respectively, Figure 3).

**Figure 4:**
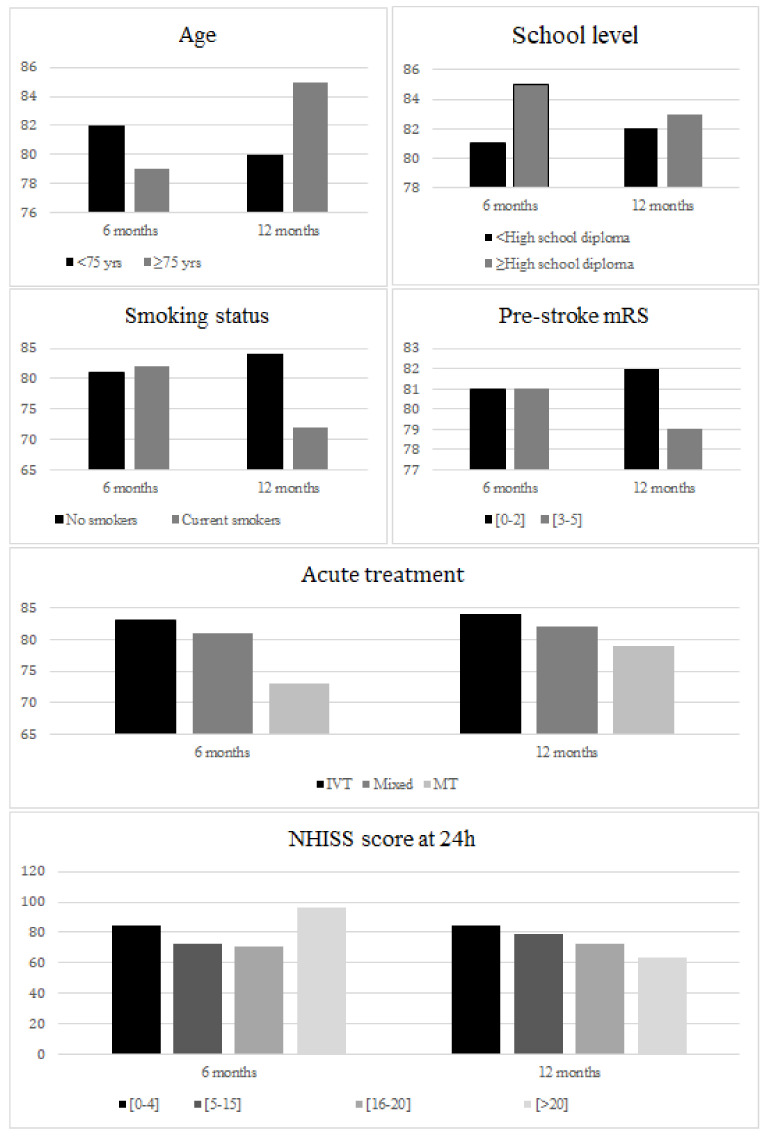
Mean mood score at 6 months and 12 months according to patients’ characteristics.

Regarding mood, the observed difference in scores narrowed significantly over time between groups defined by age ≥ 75 vs. <75 years, by NIHSS score at 24 h ≤ 4 vs. >20 (interaction *p* 0.0026 and 0.0053, respectively, Figure 4).

**Figure 5:**
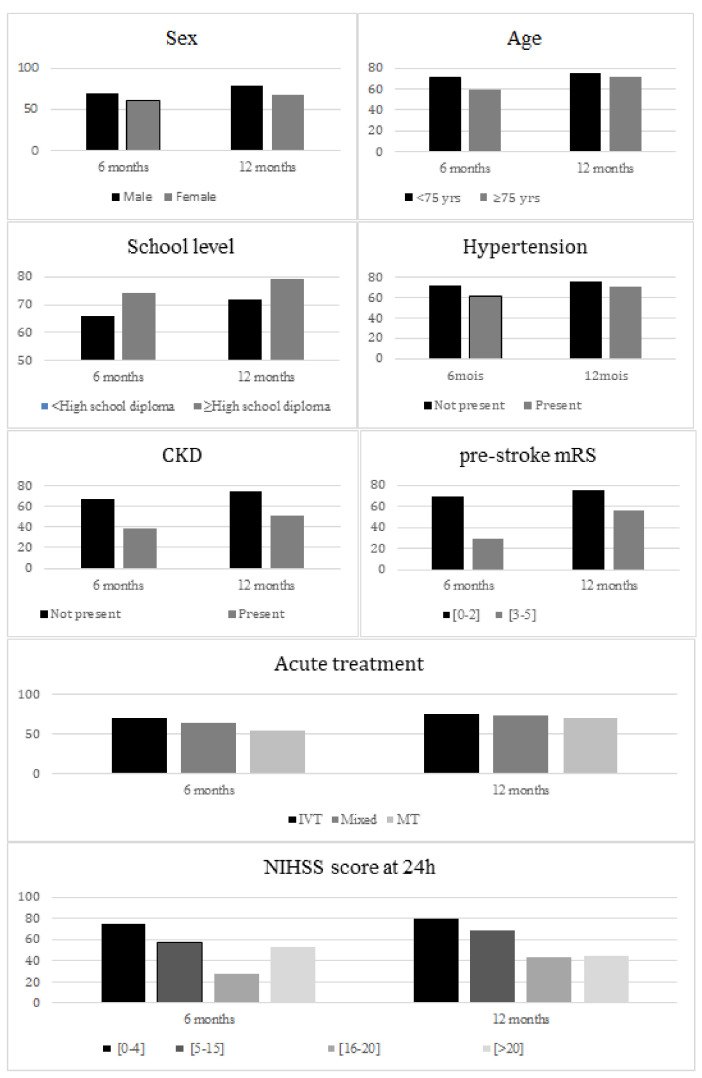
Mean social roles score at 6 months and 12 months according to patients’ characteristics. *CKD, chronic kidney disease*.

The observed difference in social roles scores narrowed significantly over time between groups defined by treatment with IVT alone vs MT alone, by a prestroke mRS score ≤ 2 vs. >2 (interaction *p* 0.0095 and 0.0016, respectively, Figure 5).

### 3.4. Multivariate Analysis

In multivariate models, there was no significant effect of time on QoL scores outside of the social roles domain. Factors associated with QoL in stroke survivors in the final multivariate model are described in Table 4 and Table 5. Factors independently associated with a lower mobility score were age > 75 years, education level below a high school diploma, peripheral arterial disease (PAD), prestroke mRS > 2, and 24-h NIHSS score > 4 (*p* < 0.0001, 0.0399, 0.0221, <0.0001, and <0.0001, respectively).

Regarding self-care, the independent associated factors were age over 75 years, school level less than high school diploma, pre-stroke mRS score > 2, and 24-h NIHSS score > 4 (*p* < 0.0001, 0.0244, 0.0006, and <0.0001, respectively). The change over time in the self-care score was modified by OSA and NIHSS at 24 h (*p* = 0.0337 and 0.0053 respectively).

Concerning the mood score, the independent factors associated were age over 75, type of treatment MT, and NIHSS score at 24 h between 5 and 15 (*p* = 0.0416, 0.03 and 0.0003 respectively). A significant interaction with time was noted for age and NIHSS score at 24 h (*p* = 0.0015 and 0.0048 respectively).

Finally, regarding the social roles domain, the independent factors associated were chronic kidney disease, pre-stroke mRS score > 2, and 24-h NIHSS score > 4 (*p* = 0.0097, <0.0001, and <0.0001, respectively). A significant interaction with time was observed for the pre-stroke mRS score (*p* = 0.0006).

Patients aged 75 years or older had a greater improvement in mobility and mood from 6 to 12 months (*p* for interaction with time: 0.0194, 0.0015 respectively), compared to younger subjects. Those with OSA had a greater improvement in self-care between 6 and 12 months (*p* for interaction with time: 0.033) compared to patients without OSA. Patients who received combined therapy had a greater improvement in mobility between 6 and 12 months (*p* for interaction with time: 0.0072), compared to those who received IVT alone. Moderate to severe pre-stroke disability (mRS > 2) was associated with a greater improvement in social roles score between 6 and 12 months (*p* for interaction with time: 0.0006) compared with subjects with a mild or no pre-stroke disability (mRS ≤ 2). Patients with NIHSS score > 20 had greater improvement in self-care than those with NIHSS scores between 0 and 4 at 24 h. Patients with NIHSS scores between 5 and 15 at 24 h had greater improvement in mood between 6 and 12 months (*p* for interaction with time: 0.0053, 0.0048, respectively) than those with mild severity (NIHSS 24 h between 0 and 4).

## 4. Discussion

To the best of our knowledge, our study is the first to describe the evolution of SS-QoL scores in the first year after ischemic stroke in patients treated with acute revascularization therapy. Depending on the domain studied, lower post-stroke QoL was independently related to lower level of school education, prestroke mRS > 2, and 24 h NIHSS score > 4. These results support previous data: the social role was the most impacted of the domains as previously described [16]. Moreover, age over 75 years [17,18], low educational level [19], high prestroke mRS (>2) [19,20,21], and NIHSS score at 24 h > 4 [22,23,24,25,26] have been reported to be associated with poor quality of life in previous studies.

Remarkably, changes in QoL scores between 6 and 12 months were not significant in multivariate analysis outside the Social Roles domain. This result is surprising, as the Social Roles domain has been reported [13] as one of the least sensitive domains to change in the SS-QoL (SRM for improvement: 0.04). This suggests that change in this domain is likely important. The lack of significant change in the Self-Care domain despite its good responsiveness [13] (SRM for improvement: 0.86) supports the hypothesis that patients’ perceptions of their ability to self-manage do not change much in the first year in our sample. This is likely due to the selection of the most autonomous patients, resulting in a ceiling effect of responses at the 6-month visit (median score: 100), as the frailest patients were unable to answer the questionnaires.

However, some variables defined different patterns of change in the QoL scores. The predictors of unfavorable evolution of QoL over time were age < 75 years (self-care, mobility, and mood domains), NIHSS score ≤ 4, (self-care and mood domains), and mRS score ≤ 2 (social roles domain). The effect of age may be explained by the better physical condition and greater autonomy of the younger patients, making it more difficult to return to the perception of the pre-stroke state. The type of revascularization therapy had no significant effect on the QoL scores, but patients treated with MT (either alone or as bridging therapy) had significantly greater improvement in Mobility score between 6 and 12 months than patients treated with IVT alone. This result could indicate a long-term beneficial effect of such a therapy on motor function. Most studies focused on the early functional outcome (at three months or six months) of patients treated with MT [5,9,27,28,29], and our findings may indicate an additional long-term benefit of MT over IVT alone. One explanation could be the fact that stroke patients eligible to MT have more severe stroke, with more frequently motor impairment, which recovery may take several months.

The results of this study provide insight into the interpretation of quality of life data in future randomized clinical trials in patients treated with revascularization. First, despite the demonstrated efficacy of revascularization on vital and functional prognosis, the change in quality of life scores between 6 and 12 months after adjustment for relevant covariates is too small to reach significance, let alone clinical relevance, in most domains studied. The expected benefit in terms of quality of life of revascularization is therefore probably moderate. On the other hand, our study showed variables with a significant effect on quality of life and its evolution between 6 and 12 months, such as age, level of education, PAD, OSA, or mRS score before stroke. Although some associations are difficult to interpret, these variables would need to be balanced between the randomization groups to allow a correct interpretation of the results in terms of quality of life.

Our study had some limitations. First, our study population may not be a representative sample of all stroke survivors. Indeed, 27.5% of the stroke survivors included in the PARADISE study were not able to complete the SS-QoL questionnaire. The subjects included in our analysis were mostly younger and less affected by the severity of the stroke. Unfortunately, this pitfall could not be avoided since the most severe patients were not able to complete the questionnaire. There is no stroke-specific quality of life scale suitable for patients with the most severe forms of aphasia or severe dementia. We sought to minimize this selection bias by administering the questionnaire by telephone to patients who were unable to attend follow-up visits. Specific information about rehabilitation process was not collected. Although all patients were managed according to standard care, including rehabilitation when indicated, we cannot exclude that differences in rehabilitation programs may have influenced patients’ recovery and QoL.

## 5. Conclusions

Quality of life evolution over one year after ischemic stroke treated by revascularization had only slight variation, thus questioning about its relevance as endpoint of future randomized clinical trials. Changes in self-care, autonomy, and mood were independently negatively affected by age younger than 75 years, and stroke severity was associated with negative changes in mood and self-care. MT (either alone or as bridging therapy) may be associated with a greater improvement in perceived mobility between 6 and 12.

## Figures and Tables

**Table 1:** Baseline characteristics of patients enrolled in the PARADISE study according to their inclusion in the QoL analysis.

	Overall * (*n* = 900)		Excluded *^,1^ (*n* = 399)		Included * (*n* = 501)		*p*-Value ^2^
**Socio-demographic characteristics**							
Female	449	(50%)	202	(51%)	247	(49%)	0.49
Age	75	(64, 83)	79	(70, 87)	70	(60, 80)	<0.001
Age ≥ 75 years old	457	(51%)	252	(63%)	205	(41%)	<0.001
**Lifestyle**							
Institution	49	(6%)	44	(11%)	5	(1%)	<0.001
individual housing	840	(94%)	347	(89%)	493	(99%)	
**School level**							
Higher than High school diploma	163	(26%)	40	(20%)	123	(29%)	0.016
**Employement status**							
Unemployed	88	(11%)	31	(10%)	57	(12%)	<0.001
Employed	131	(17%)	27	(9%)	104	(22%)	
Retired	563	(72%)	249	(81%)	314	(66%)	
**Medical History**							
18.5 ≤ BMI ≤ 25	273	(30%)	95	(24%)	178	(36%)	<0.001
BMI > 25	386	(43%)	137	(34%)	249	(50%)	
BMI < 18.5	241	(27%)	167	(42%)	74	(15%)	
Hypertension	560	(63%)	275	(71%)	285	(58%)	<0.001
Diabetes	136	(15%)	81	(21%)	55	(11%)	<0.001
Hypercholesterolemia	298	(34%)	129	(34%)	169	(34%)	0.74
Active smoker	157	(18%)	50	(13%)	107	(22%)	<0.001
Coronary artery disease	117	(13%)	62	(16%)	55	(11%)	0.043
OSA	78	(9%)	44	(11%)	34	(7%)	0.005
PAD	36	(4%)	26	(7%)	10	(2%)	0.002
AF	319	(36%)	191	(49%)	128	(26%)	<0.001
Cancer	135	(15%)	66	(17%)	69	(14%)	0.13
CKD	49	(6%)	31	(8%)	18	(4%)	0.010
Previous Stroke	113	(13%)	62	(16%)	51	(11%)	0.028
**Stroke location**							0.36
Anterior circulation	725	(85%)	326	(86%)	399	(84%)	
Posterior circulation	128	(15%)	52	(14%)	76	(16%)	
**Acute stroke treatment**							<0.001
IVT	425	(48%)	155	(39%)	270	(55%)	
Mixed	176	(20%)	72	(18%)	104	(21%)	
MT	292	(33%)	171	(43%)	121	(24%)	
**Glasgow score**							<0.001
15	662	(74%)	269	(68%)	393	(78%)	
[9–14]	89	(10%)	55	(14%)	34	(7%)	
<9	147	(16%)	73	(18%)	74	(15%)	
**Handicap scores and stroke severity**							
Pre-stroke mRS	0	(0, 1)	0	(0, 3)	0	(0, 0)	<0.001
NIHSS at admission	11	(6, 18)	16	(9, 20)	9	(5, 15)	<0.0001
NIHSS at 24 h	6	(2, 14)	13	(6, 20)	4	(1, 8)	<0.001

^1^ Patients were excluded of the present study due to missing data for quality of life measure at both time points (6 and 12 month post stroke). ^2^ Comparison between included and excluded subjects using chi-square (qualitative variables) or Mann-Whitney test (quantitative variables). * Median (IQR) or number (percentage). IQR, inter quartile range; OSA, obstructive sleep apnea; PAD, peripheral artery disease; AF, atrial fibrillation, CKD, chronic kidney disease.

**Table 2:** SS-QoL quality of life scores at 6 and 12 months.

	6 Month			12 Month			Evolution from 6 to 12 Months	
	*n*	Median	(IQR)	*n*	Median	(IQR)	Mean	±Std
**Self-care**	408	100	(80, 100)	368	100	(95, 100)	+1.83	±13.6
**Mobility**	407	87	(54, 100)	367	92	(63, 100)	+1.87	±17.7
**Mood**	403	90	(70, 100)	368	95	(70, 100)	−0.26	±21
**Social role**	403	80	(31, 100)	367	90	(50, 100)	+3.80	±28.7

The score in each domain was transformed into a score from 0 (worst QoL) to 100 (best possible QoL). IQR, interquartile range; std, standard deviation.

**Table 3:** Bivariate association between initial patient characteristics and SS-QoL outcome adjusted on time.

	Self-Care			Mobility			Mood			Social Roles		
	Estimate ^1^	*p*-Value ^2^	Time Interaction ^3^	Estimate ^1^	*p*-Value ^2^	Time Interaction ^3^	Estimate ^1^	*p*-Value ^2^	Time Interaction ^3^	Estimate ^1^	*p*-Value ^2^	Time Interaction ^3^
**Sex** Male	87.00	-		78.90	-		80.97	-		69.51	-	
Female	80.27	0.0112	NS	66.42	<0.0001	NS	79.59	0.5758	NS	61.00	0.0196	NS
**Age** < 75 yrs	89.45	-		80.65	-		82.09	-		70.34	-	
≥75yrs	75.46	<0.0001	0.0189	61.32	<0.0001	0.0464	77.57	0.071	0.0026	58.42	0.0007	NS
**School level** < High school	82.71	-		71.61	-		80.96	-		64.95	-	
≥High school	92.74	0.0006	NS	83.09	0.0006	NS	83.83	0.2978	NS	73.69	0.0341	NS
**BMI** 18.5 ≤ BMI ≤ 25	85.84	-		74.49	-		81.38	-		63.53	-	
BMI > 25	84.20	0.5679	NS	73.40	0.7343	NS	81.20	0.9452	NS	67.99	0.2540	NS
BMI < 18.5	76.50	0.0247	NS	65.42	0.0524	NS	73.85	0.0572	NS	60.83	0.6414	NS
**Hypertension** No	88.65	-		79.39	-		81.18	-		72.10	-	
Yes	80.21	0.0016	NS	67.98	0.0001	NS	79.26	0.4459	NS	60.23	0.0011	NS
**Diabetes** No	83.87	-		73.54			79.84	-		66.92	-	
Yes	81.08	0.512	NS	66.38	0.1271	NS	80.03	0.9621	NS	56.73	0.0735	NS
**CAD** No	84.32	-		73.35	-		80.28	-		66.16	-	
Yes	79.04	0.2056	NS	68.36	0.2850	NS	80.18	0.9793	NS	59.45	0.2327	NS
**OSA** No	84.49	-		73.45	-		80.84	-		66.32	-	
Yes	73.54	0.0357	0.0229	62.76	0.0702	NS	72.77	0.1027	NS	57.06	0.1901	NS
**AF** No	86.16	-		75.02	-		81.04	-		65.85	-	
Yes	76.67	0.0017	NS	64.74	0.0024	NS	77.65	0.2332	NS	64.09	0.6675	NS
**Previous Stroke** No	84.68	-		73.38	-		80.50	-		67.01	-	
Yes	75.08	0.0286	NS	65.25	0.0951	NS	76.41	0.3215	NS	55.20	0.0434	NS
**PAD** No	83.52	-		72.57	-		79.57	-		65.76	-	
Yes	82.39	0.9077	NS	49.97	0.0372	NS	85.43	0.5304	NS	49.20	0.2154	NS
**CKD** No	84.11	-		73.22	-		80.41	-		66.51	-	
Yes	68.70	0.0312	NS	52.45	0.0091	NS	71.45	0.1827	NS	34.35	0.0009	NS
**Cancer** No	83.26	-		72.98	-		80.42	-		65.92	-	
Yes	85.29	0.5959	NS	69.90	0.4702	NS	78.05	0.5060	NS	61.07	0.3483	NS
**Acute treatment** IVT	87.13	-		77.40	-		83.34	-		71.27	-	
Combined	84.20	0.3847	NS	71.73	0.130	0.0484	80.77	0.4132	NS	63.34	0.0749	NS
MT	76.17	0.0007	NS	62.95	<0.0001	NS	73.41	0.0011	NS	53.01	<0.0001	0.0095
**Pre-stroke mRS** [0–2]	85.50	-		75.15	-		80.33	-		68.21	-	
[3–5]	62.41	<0.0001	NS	44.46	<0.0001	NS	81.12	0.8741	NS	28.28	<.0001	0.0016
**NIHSS at 24h** [0–4]	94.57	-		80.23	-		84.63	-		74.40	-	
[5–15]	76.99	<0.0001	0.0002	68.60	0.0001	NS	73.15	<0.0001	NS	57.70	<0.0001	NS
[16–20]	43.68	<0.0001	NS	46.40	<0.0001	NS	69.55	0.0086	NS	27.75	<0.0001	NS
[>20]	36.20	<0.0001	NS	30.87	<0.0001	NS	93.26	0.289	0.0053	56.18	0.1157	NS

^1^ Quality of life in the presented category of the variable, as estimated by mixed model taking into account M6 and M12 measure, adjusted on time effect (M6 or M12) and on time * variable interaction. ^2^ A significant effect for the considered variables indicates a significant difference in QoL scores across levels of the variable, taking into account M6 and M12 measures. A dash indicates the reference category. ^3^ A significant time * variable interaction an increase (or a decrease) in the QoL difference between the two levels of the variables between 6 and 12 months. BMI, body mass index; CAD, coronary artery disease; OSA, obstructive sleep apnea; AF, atrial fibrillation; CKD, chronic kidney diseases; PAD, peripheral arterial disease.

**Table 4:** Multivariate analysis of characteristics associated with the SS-QoL domains self-care and mobility.

Variable		Estimate ^1^	Standard Error	*p*-Value for Variable Effect ^2^	*p*-Value for Time * Variable Interaction ^3^
	**SS-QoL Self-Care**				
	Intercept ^4^	98.79	1.94		-
**Period**	12 months (vs 6 months)	−1.15	1.33	0.3879	-
**Age**	≥75 yrs	−9.51	2.33	<0.0001	0.0853
**School level**	≥High school	+4.86	2.15	0.0244	NT
**Anterior stroke**	Yes	+4.24	3.21	0.1873	NT
**OSA**	Yes	−8.19	4.29	0.0570	0.0337
**Pre-stroke mRS**	[3–5] (vs. [0–2])	−13.39	3.88	0.0006	NT
**24 h NIHSS score** (*ref [0–4]*)	[5–15]	−14.53	2.37	<0.0001	0.0053
	[16–20]	−46.77	6.30	<0.0001	NT
	[>20]	−56.56	7.63	<0.0001	NT
	**SS-QoL Mobility**				
	Intercept ^4^	90.00	2.87	<0.0001	
**Period**	12 months (vs. 6 months)	−2.20	1.82	0.2281	-
**Sex**	Female	−4.52	2.63	0.0860	NT
**Age**	>75 yrs	−13.45	2.99	<0.0001	0.0194
**School level**	≥High school	+5.99	2.90	0.0399	NT
**PAD**	Yes	−19.47	8.47	0.0221	NT
**Acute treatment** (*ref: IVT*)	Mixed	−6.62	3.61	0.0678	0.0072
	MT	−7.09	3.76	0.0602	0.0875
**Pre-Stroke mRS**	[3–5] (vs. [0–2])	−20.84	5.20	<0.0001	NT
**24 h NIHSS score** (*ref [0–4]*)	[5–15]	−6.55	2.84	0.0217	NT
	[16–20]	−33.69	6.36	<0.0001	NT
	[>20]	−41.34	8.49	<0.0001	NT

^1^ Mean QoL score difference between presented category of the variable, and reference category, as estimated by mixed model, adjusted on the effect of the covariates presented in the table and time * variable interactions, with a backward procedure for variable selection and a 0.10 threshold for variable exit. ^2^ A significant effect for the considered variable indicates an independent significant difference in QoL scores across levels of the variable. ^3^ A significant time * variable indicates interaction an increase (or a decrease) in the QoL difference between the two levels of the variables between 6 and 12 months. ^4^ The estimate for the intercept is the quality of life score, as estimated by mixed model, for a patient being in the reference category for all covariates included in the final model. NT, not tested (i.e., not retained by the variable selection procedure); PAD, peripheral artery disease; IVT, intravenous thrombolysis; MT, mechanical thrombectomy; mRS, modified ranking scale; NIHSS, National Institutes of Health Stroke Scale Score.

**Table 5:** Multivariate analysis of characteristics associated with SS-QoL domains mood and social roles.

Variable		Estimate ^1^	Standard Error	*p*-Value for Variable Effect ^2^	*p*-Value for time * variable Interaction ^3^
	**SS-QoL Mood**				
	Intercept	88.07	2.10	<0.0001	
**Period**	12 months (vs. 6 months)	−3.65	1.86	0.0510	-
**Age**	≥75 yrs	−5.14	2.51	0.0416	0.0015
**Acute treatment** *(ref IVT)*	Mixed	−2.35	2.81	0.4027	NT
	MT	−6.02	2.77	0.030	NT
**24 h NIHSS score** *(ref [0–4]*)	[5–15]	−9.83	2.68	0.0003	NT
	[16–20]	−9.75	5.93	0.1008	NT
	[>20]	+11.45	8.17	0.1615	0.0048
	**SS-QoL Social roles**				
	Intercept	78.67	2.93	<.0001	-
**Period**	12 months (vs. 6 months)	+3.47	1.71	0.0427	-
**Sex**	Female	−5.13	3.02	0.0907	NT
**School level**	≥High school	+5.92	3.36	0.0784	NT
**Chronic kidney disease**	Yes	−21.54	8.29	0.0097	NT
**Pre- Stroke mRS**	[3–5] (vs. [0–2])	−37.93	7.33	<0.0001	0.0006
**24 h NIHSS score** (*ref [0–4]*)	[5–15]	−14.53	3.22	<0.0001	NT
	[16–20]	−37.73	7.37	<0.0001	NT
	[>20]	−24.44	10.18	0.0168	NT

^1^ Mean QoL score difference between presented category of the variable, and reference category, as estimated by mixed model, adjusted on the effect of the covariates presented in the table and time * variable interactions, with a backward procedure for variable selection and a 0.10 threshold for variable exit. ^2^ A significant effect for the considered variable indicates an independent significant difference in QoL scores across levels of the variable. ^3^ A significant time * variable interaction indicates an increase (or a decrease) in the QoL difference between the two levels of the variables between 6 and 12 months. ^4^ The estimate for the intercept is the quality of life score, as estimated by mixed model, for a patient being in the reference category for all covariates included in the final model. NT, not tested (i.e., not retained by the variable selection procedure); IVT, intravenous thrombolysis; MT, mechanical thrombectomy; mRS, modified ranking scale; NIHSS, National Institutes of Health Stroke Scale Score. Changes in mobility score over time type was significantly influenced by age and the type of revascularization therapy (*p* for interaction with time: 0.0194, and 0.0072, respectively); patients treated with combined therapy had a significantly greater improvement between 6 and 12 month compared with those who received IVT alone.

## Data Availability

The authors declare that all supporting data are available within the article.

## References

[1] Lecoffre C., de Peretti C., Gabet A., Grimaud O., Woimant F., Giroud M., Béjot Y., Olié V. (2017). National Trends in Patients Hospitalized for Stroke and Stroke Mortality in France, 2008 to 2014. Stroke.

[2] Guéniat J., Brenière C., Graber M., Garnier L., Mohr S., Giroud M., Delpont B., Blanc-Labarre C., Durier J., Giroud M. (2018). Increasing Burden of Stroke: The Dijon Stroke Registry (1987–2012). Neuroepidemiology.

[3] Gabet A., Grimaud O., de Peretti C., Béjot Y., Olié V. (2019). Determinants of Case Fatality After Hospitalization for Stroke in France 2010 to 2015. Stroke.

[4] Rosso C., Blanc R., Ly J., Samson Y., Lehéricy S., Gory B., Marnat G., Mazighi M., Consoli A., Labreuche J. (2019). Impact of Infarct Location on Functional Outcome Following Endovascular Therapy for Stroke. J. Neurol. Neurosurg. Psychiatry.

[5] Froehler M.T., Saver J.L., Zaidat O.O., Jahan R., Aziz-Sultan M.A., Klucznik R.P., Haussen D.C., Hellinger F.R., Yavagal D.R., Yao T.L. (2017). Interhospital Transfer Before Thrombectomy Is Associated With Delayed Treatment and Worse Outcome in the STRATIS Registry (Systematic Evaluation of Patients Treated With Neurothrombectomy Devices for Acute Ischemic Stroke). Circulation.

[6] Gunaydin R., Karatepe A.G., Kaya T., Ulutas O. (2011). Determinants of Quality of Life (QoL) in Elderly Stroke Patients: A Short-Term Follow-up Study. Arch. Gerontol. Geriatr..

[7] Emberson J., Lees K.R., Lyden P., Blackwell L., Albers G., Bluhmki E., Brott T., Cohen G., Davis S., Donnan G. (2014). Effect of Treatment Delay, Age, and Stroke Severity on the Effects of Intravenous Thrombolysis with Alteplase for Acute Ischaemic Stroke: A Meta-Analysis of Individual Patient Data from Randomised Trials. Lancet.

[8] Goyal M., Menon B.K., van Zwam W.H., Dippel D.W.J., Mitchell P.J., Demchuk A.M., Dávalos A., Majoie C.B.L.M., van der Lugt A., de Miquel M.A. (2016). Endovascular Thrombectomy after Large-Vessel Ischaemic Stroke: A Meta-Analysis of Individual Patient Data from Five Randomised Trials. Lancet.

[9] Campbell B.C.V., Donnan G.A., Lees K.R., Hacke W., Khatri P., Hill M.D., Goyal M., Mitchell P.J., Saver J.L., Diener H.-C. (2015). Endovascular Stent Thrombectomy: The New Standard of Care for Large Vessel Ischaemic Stroke. Lancet Neurol..

[10] Ramos-Lima M.J.M., Brasileiro I.d.C., de Lima T.L., Braga-Neto P. (2018). Quality of Life after Stroke: Impact of Clinical and Sociodemographic Factors. Clinics.

[11] Silva S.M., Corrêa F.I., de Morais Faria C.D.C., Corrêa J.C.F. (2015). Psychometric Properties of the Stroke Specific Quality of Life Scale for the Assessment of Participation in Stroke Survivors Using the Rasch Model: A Preliminary Study. J. Phys. Ther. Sci..

[12] Pan J.H., Song X.Y., Lee S.Y., Kwok T. (2008). Longitudinal Analysis of Quality of Life for Stroke Survivors Using Latent Curve Models. Stroke.

[13] Legris N., Devilliers H., Daumas A., Carnet D., Charpy J.P., Bastable P., Giroud M., Bejot Y. (2018). French Validation of the Stroke Specific Quality of Life Scale (SS-QoL). NeuroRehabilitation.

[14] Williams L.S., Redmon G., Saul D.C., Weinberger M. (2001). Reliability and Telephone Validity of the Stroke-Specific Quality of Life (SS-QOL) Scale. Stroke.

[15] Caël S., Decavel P., Binquet C., Benaim C., Puyraveau M., Chotard M., Moulin T., Parratte B., Bejot Y., Mercier M. (2015). Stroke Impact Scale Version 2: Validation of the French Version. Phys. Ther..

[16] Obembe A.O., Eng J.J. (2016). Rehabilitation Interventions for Improving Social Participation After Stroke: A Systematic Review and Meta-Analysis. Neurorehabil. Neural Repair.

[17] Panício M.I., Mateus L., Ricarte I.F., de Figueiredo M.M., Fukuda T.G., Seixas J.C., Ferraz M.E., Silva G.S. (2014). The Influence of Patient’s Knowledge about Stroke in Brazil: A Cross Sectional Study. Arq. Neuro-Psiquiatr..

[18] Pontes-Neto O.M., Silva G.S., Feitosa M.R., De Figueiredo N.L., Fiorot Júnior J.A., Rocha T.N., Massaro A.R., Leite J.P. (2008). Stroke Awareness in Brazil—Alarming Results in a Community-Based Study. Stroke.

[19] Ahlsiö B., Britton M., Murray V., Theorell T. (1984). Disablement and Quality of Life after Stroke. Stroke.

[20] Opara J.A., Jaracz K. (2010). Quality of Life of Post-Stroke Patients and Their Caregivers. J. Med. Life.

[21] Szőcs I., Dobi B., Lám J., Orbán-Kis K., Häkkinen U., Belicza É., Bereczki D., Vastagh I. (2020). Health Related Quality of Life and Satisfaction with Care of Stroke Patients in Budapest: A Substudy of the EuroHOPE Project. PLoS ONE.

[22] Christensen M.C., Mayer S., Ferran J.-M. (2009). Quality of Life after Intracerebral Hemorrhage: Results of the Factor Seven for Acute Hemorrhagic Stroke (FAST) Trial. Stroke.

[23] Sturm J.W., Donnan G.A., Dewey H.M., Macdonell R.A.L., Gilligan A.K., Srikanth V., Thrift A.G. (2004). Quality of Life after Stroke: The North East Melbourne Stroke Incidence Study (NEMESIS). Stroke.

[24] Ronne-Engström E., Enblad P., Lundström E. (2011). Outcome after Spontaneous Subarachnoid Hemorrhage Measured with the EQ-5D. Stroke.

[25] Sprigg N., Selby J., Fox L., Berge E., Whynes D., Bath P.M.W. (2013). Very Low Quality of Life After Acute Stroke. Stroke.

[26] Fischer U., Anca D., Arnold M., Nedeltchev K., Kappeler L., Ballinari P., Schroth G., Mattle H.P. (2008). Quality of Life in Stroke Survivors after Local Intra-Arterial Thrombolysis. Cerebrovasc. Dis..

[27] Campbell B.C.V., Mitchell P.J., Churilov L., Keshtkaran M., Hong K.-S., Kleinig T.J., Dewey H.M., Yassi N., Yan B., Dowling R.J. (2017). Endovascular Thrombectomy for Ischemic Stroke Increases Disability-Free Survival, Quality of Life, and Life Expectancy and Reduces Cost. Front. Neurol..

[28] Nogueira R.G., Lutsep H.L., Gupta R., Jovin T.G., Albers G.W., Walker G.A., Liebeskind D.S., Smith W.S. (2012). TREVO 2 Trialists Trevo versus Merci Retrievers for Thrombectomy Revascularisation of Large Vessel Occlusions in Acute Ischaemic Stroke (TREVO 2): A Randomised Trial. Lancet.

[29] Kumar G., Shahripour R.B., Alexandrov A.V. (2015). Recanalization of Acute Basilar Artery Occlusion Improves Outcomes: A Meta-Analysis. J. Neurointerv. Surg..

